# Symbiont retention and holobiont response under simulated sulfide deprivation in Lucinid clams from seagrass beds

**DOI:** 10.3389/fmicb.2025.1637201

**Published:** 2025-11-18

**Authors:** Samuel Orgeas-Gobin, Bérénice Piquet, Benjamin Marie, Ann C. Andersen, Arnaud Tanguy, Sébastien Duperron

**Affiliations:** 1 UMR 7144 AD2M Adaptation et Diversité en Milieu Marin, Sorbonne Université, Station Biologique de Roscoff, Roscoff, France; 2 UMR 7245 MCAM, Molécules de Communication et Adaptation des Microorganismes, Muséum national d'Histoire naturelle, Paris, France

**Keywords:** chemosynthesis, seagrass bed, *Lucinoma borealis*, *Loripes orbiculatus*, *Zostera*

## Abstract

Bivalves of the family Lucinidae thrive in sulfidic sediments thanks to their chemoautotrophic bacterial symbionts. However, how different Lucinidae species respond to sulfide deprivation and associated symbiont loss remains poorly understood. Here, we investigated the responses of *Lucinoma borealis* and *Loripes orbiculatus*, two species that co-occur in temperate seagrass beds, exposed to prolonged sulfide starvation. Using metabolomics, ultrastructural TEM analyses and 16S rRNA-based metabarcoding, we monitored and compared responses in gills and visceral mass over a 4-month period. Both host species as well as their symbionts survived sulfide-free conditions. Hosts tissues displayed limited impact on ultrastructure and metabolites. Despite decrease in numbers and activity level, symbionts remained present throughout the experiment and no evidence for bacteremia or infection was detected. Our results also revealed differences, in particular in host apoptosis response, suggesting species-specific stress strategies. Altogether, both holobionts can survive extended low-sulfide periods without critical damage and without completely losing their symbionts. These could be adaptations to the extended low-sulfide periods that are associated with low primary production and the cold season in seagrass beds. Adaptations could involve a switch in the symbionts' physiological state to preserve a dormant symbiotic population. These findings highlight the importance of stress tolerance mechanisms in coastal Lucinidae, and raise questions about the nature of host–symbiont dependency in these periods.

## Introduction

Symbiotic relationships between Lucinidae (Bivalvia) and sulfur-oxidizing autotrophic bacteria are estimated to have established between 250 to 465 million years ago (Taylor and Glover, 2006; Sanchez et al., 2007; Brissac et al., 2009; Stanley, 2014). Lucinids represent the most diverse known family of chemosymbiotic bivalves, both in terms of species as well as diversity of habitats where they occur, which range from coastal waters to the deep sea worldwide (Taylor and Glover, 2006; Roeselers and Newton, 2012). Their associated bacteria perform CO_2_ fixation thanks to energy obtained from the aerobic oxidation of reduced sulfur compounds, and contribute to the host's nutrition by transferring organic compounds derived from chemosynthesis (Petersen et al., 2016; Ratinskaia et al., 2024).

Among other habitats, lucinids colonize anoxic sediment zones under seagrass meadows, where fermentation of organic matter from seagrass roots and infauna by sulfate-reducing bacteria locally generates high sulfide concentrations. A tripartite symbiosis has been proposed between Lucinidae, their sulfur-oxidizing gill bacteria, and seagrass plants. In this model, the Lucinidae-sulfide oxidizer symbiosis oxidizes H_2_S, a molecule toxic to most multicellular organisms, and by reducing sulfidic stress, enhances seagrass growth and productivity (van der Heide et al., 2012). In temperate seagrass beds, besides the sulfidic stress, strong cyclical environmental fluctuations occur at various temporal scales—hourly (e.g., tides and currents), episodic (e.g., anthropogenic inputs), or seasonal. These changes, in particular seasonal changes in temperature and light availability, influence seagrass productivity and activity of microbial degraders in the sediments. These can lead to fluctuations in sulfide production and availability. During winter, colder temperatures, reduced primary production and bacterial activity may result in low sulfide concentrations, raising questions about the role of chemosynthetic symbiosis in lucinids and the fate of symbionts in host gills. Whether symbionts are maintained during unfavorable conditions, or eliminated until sulfide becomes available again is not known. Indeed, to date, most studies have focused either on tropical lucinid species inhabiting mangrove sediments—environments with relatively stable tidal and temperature regimes—or deep-sea species, where host–symbiont interactions are highly specialized and tightly adapted to very different constraints. While informative, these examples may not accurately reflect the environmental variability experienced by temperate coastal lucinids.

*Lucinoma borealis* (Codakiinae, Linnaeus, 1767) and *Loripes orbiculatus* (Lucininae, Poli, 1791) co-occur in *Zostera marina* seagrass meadows in the Bay of Roscoff (Brittany, France). *Lu. borealis* is distributed over a wide geographic range, extending across the Northern Atlantic, from Norway to the Mediterranean Sea, and around Britain and Ireland. It inhabits sandy sediments from intertidal zones down to 200 meters depth on the continental shelf (Dando et al., 1986; Duperron et al., 2013). *Lo. orbiculatus* also occurs in various environments, including the North Sea, the Northeast Atlantic, along the African coasts, in tropical waters, as well as in the Mediterranean and the Black Sea, at depths ranging from coastal areas to approximately 150 meters (Yuen et al., 2012). These two species are frequently associated with seagrass meadows (*Zostera* spp). Because both species co-occur and harbor Gram-negative endosymbiotic sulfur-oxidizing autotrophic Gammaproteobacteria identified as *Candidatus* Thiodiazotropha sp. (Dando et al., 1986; Martin et al., 2020) located inside specialized gill epithelial cells named bacteriocytes (Fisher, 1990; Brissac et al., 2009), they are interesting models to test for potential adaptation to episodes of low sulfide availability. While several studies have demonstrated that some coastal Lucinidae are able to survive for extended periods without sulfide (Caro et al., 2009; Alcaraz et al., 2024), no recent data are available for *Lu. borealis*. The co-occurrence of *Lu. borealis* and *Lo. orbiculatus* offers an opportunity to compare their responses under similar experimental setup, where both are subjected to identical sulfide starvation conditions. Based on previous findings in other Lucinidae, we hypothesize that both species will be able to survive the experiment, but may also exhibit species-specific responses. In this study, the response of *Lu. borealis* and *Lo. orbiculatus* to sulfide starvation is investigated using an experimental approach. We simulated low sulfide conditions by maintaining individuals in sulfide-free aquaria for 4 months, thereby depriving the symbionts of their energy source. Changes in host gill ultrastructure and symbiont densities are investigated using transmission electron microscopy (TEM). The composition of gill- and visceral mass-associated bacterial communities is monitored using 16S rRNA gene sequencing and symbiont activities are monitored using qPCR. Holobiont overall metabolite composition is monitored using untargeted metabolomics as a proxy of functional changes. Finally, we monitored the expression levels of apoptosis marker genes in gill tissue using qPCR. Altogether, these data provide novel insights into the dynamics of host–symbiont relationships during extended sulfide deprivation, and the combination of approaches provides an integrated view of holobionts' response.

## Materials and methods

### Sample collection and preparation

To cope with biological issue and possible specimen loss, a total of 381 *Loripes orbiculatus* and 221 *Lucinoma borealis* specimens were collected in October 2022 from a sublittoral zone near a *Zostera marina* seagrass bed in front of the Roscoff Biological Station (Roscoff, France; 48°43′50′′N, 3°59′51′′W; depth: 20 cm). Upon collection, ten individuals were dissected under sterile conditions (control specimens at T0). The two hemibranchs and remaining visceral mass were flash-frozen separately in liquid nitrogen and stored at −80 °C (T0 samples). The first hemibranch of each individual was halved, with one half used for DNA extraction and the other for RNA extraction. The remaining pool of individuals was transferred and maintained in flow-through aquariums at the Roscoff Marine Biological Research Center (CRBM). They were maintained in natural seawater taken from Roscoff Bay that was H_2_S-free, filtered at 100 μm, and kept at room temperature with a natural day/light cycle. For each species, ten individuals were sampled at regular intervals (15, 30, 45, 60, 90, and 120 days after transfer), dissected under sterile conditions, and processed for subsequent electron microscopy, metabarcoding, qPCR, and metabolomics analyses. During the sulfide starvation experiment, a total of 120 Lucinidae specimens was used.

### DNA extraction

Gills and visceral mass were subjected to enzymatic lysis using proteinase K and lysozyme for 3 h at 59 °C. RNA was removed with RNase incubation at 37 °C for 15 min. DNA extraction followed a classical phenol–chloroform–isoamyl alcohol (25:24:1) protocol (Sambrook and Russell, 2006), and DNA quality and quantity were assessed using a NanoDrop spectrophotometer (NanoDrop One UV-Vis, Thermo Fisher Scientific, Waltham, USA).

### RNA extraction

RNA extraction was conducted using the EZNA^®^ HP Total RNA Isolation Kit (OMEGA, Norcross, GA) RNA extraction was conducted using the EZNA^®^ HP Total RNA Isolation Kit (OMEGA, Norcross, GA) according to the manufacturer's instructions. RNA quantity and quality were checked using a NanoDrop spectrophotometer (NanoDrop One UV-Vis, Thermo Fisher Scientific, Waltham, USA) using 260/280 and 230/260 nm ratios. An additional precipitation step was performed by adding 350 μL molecular-grade water, 40 μL of 7 M ammonium acetate, and 100% ethanol, followed by overnight precipitation at −20 °C to remove residual salts. Integrity of the RNA was checked by 1% agarose gel electrophoresis.

### RT-PCR and qPCR analysis

To monitor the symbiont quantity and activity during the depuration process, we amplified the 16S rRNA-encoding gene from symbionts from total DNA and total RNA (see below) in the gill tissues, and a fragment of the 18S rRNA-encoding gene from the respective host was used as an internal PCR control. Primers for genes involved in both extrinsic and intrinsic apoptotic pathways were determined from transcriptomic sequences available (primer sequences are summarized in Supplementary Table S1). All gene expressions were normalized using host 18S rRNA gene PCR as a reference. Total RNA was reverse transcribed using the M-MLV Reverse Transcriptase kit (PROMEGA, Madison, USA). After RT-PCR, cDNA was diluted (1:100) before qPCR. Relative quantification of gene expression was performed by qPCR with specific primer pairs designed directly based on the *Lucinidae* draft genome using Primer3 (v4.0.0; Untergasser et al., 2012). Primers were synthesized by Eurogentec (Seraing, Belgium). The qPCR reactions (5 μL final volume) included 2.5 μL LightCycler^®^ 480 SYBR^®^ Green I Master Mix (Roche, Mannheim, Germany), 0.1 μL primers (10 pM), and 2.3 μL template DNA, and were run on a LightCycler^®^ 480 (Roche, Mannheim, Germany) with the following parameters: initial denaturation at 95 °C, amplification at 60 °C, elongation at 72 °C for 55 cycles, followed by a melting curve analysis. Data were processed using LightCycler^®^ 480 SW 1.5.1 software. The apoptotic response of both species was monitored over the course of starvation using quantitative PCR (qPCR), by quantifying the expression levels of genes involved in the extrinsic (FAS, TNF, CASP8, CASP7, CASP3) and intrinsic (BAX, DIABLO, COX1, CASP9, CASP7, CASP3) apoptotic pathways. Data were normalized using the ΔΔCt method (Bustin et al., 2009). Following qPCR, data are expressed using the RQ (relative quantification) method. In this article, all statistical analyses were conducted in R (R Core Team, 2024). To test the effect of time on the symbiotic density and activities, Kruskal-Wallis and Dunn *post-hoc* (*p*_*adj*_ FDR) tests were used, as the data did not comply with the homoscedasticity condition (normality and homoscedasticity were tested with Shapiro and Levene tests, respectively). To test the effect of time, species and the interaction of these two terms on the symbiotic density and activities, a PERMANOVA test was used included in the R *vegan* package.

### Transmission electron microscopy

Hemibranchs from three individuals sampled at 0, 30, and 120 days of sulfide deprivation were dissected under sterile seawater to prevent desiccation. Thin slices were fixed in 4% glutaraldehyde (pH 7.4; 10% NaCl; 2.5 mM CaCl_2_; 0.4 M cacodylate) overnight at 4 °C. Samples were rinsed twice in 0.2 M cacodylate buffer (pH 7.4, 2.1% NaCl), post-fixed in 1% osmium tetroxide for 1 h, and dehydrated in graded ethanol series (35%−100%). Tissue impregnation with Spurr resin occurred in resin–ethanol mixtures (1:3, 1:2, 2:3 volume-to-volume ratios) for 1.5 heach, followed by three pure resin baths. Polymerization was performed at 60 °C for 48 h. Ultrathin 60 nm sections were cut using an ultramicrotome (RMC Boeckeler PowerTome XL, Tucson, USA), stained with uranyl acetate saturated in 70% Fsignificanywere analyzed using FIJI (Schindelin et al., 2012). Post-processing of electron microscopy images was performed using Adobe Photoshop to eliminate background noise and obvious (non-sample-related) impurities. Adjustments were limited to cosmetic corrections, ensuring that no structural or analytical information was altered. Differences in cell counts and diameter were tested using Kruskal-Wallis and Dunn *post-hoc* (*p*_*adj*_ FDR) tests (homoscedasticity hypothesis was rejected by a Levene test).

### Sequential metabolites and DNA extraction

For both species, three hemibranchs and three visceral masses were sampled at 0, 15, 45, 60, 90, and 120 days of depuration, dissected under sterile conditions, flash-frozen, and stored at −80 °C. Sequential metabolite and DNA extractions were performed to analyze the metabolome and microbiome of the same individual sample following the protocol described in Duperron et al. (2023). In short, samples were suspended in 200 μL of cold UHPLC-grade methanol–water (75:25%), homogenized mechanically (GLH850 OMNI, 25,000 rpm, 30 s), and sonicated (Sonics Vibra-Cell VCX 130, 60% amplitude, 30 s) on ice. Supernatants collected post-centrifugation (15,300 g, 4 °C, 10 min) were stored in amber vials at −20 °C for LC/MS analysis (see below). DNA was then extracted from pellets using the QIAGEN PowerLyzer PowerSoil DNA Kit (Hilde, Germany) following the manufacturer's instructions and using FastPrep 5G disruption (5 × 30 s, 8 m/s). An extraction blank was included.

### Metabolite analysis

Metabolites from the gills and visceral mass of *Lu. borealis* and *Lo. orbiculatus* were analyzed using ultra-high-performance liquid chromatography (UHPLC; ELUTE, Bruker) coupled with high-resolution mass spectrometry (ESI-Qq-TOF Compact, Bruker). For each sample, 2 μL of extract were injected and separated using a Polar Advance II C18 column (2.5 μm pore size, Thermo Fisher) at a flow rate of 300 μL/min under a linear gradient of 0.1% formic acid in acetonitrile (5–90% over 15 min). Electrospray ionization (ESI) parameters were: capillary temperature 200 °C, source voltage 3.5 kV, and gas flow 8 L/min. Ions (50–1,500 m/z) were detected in positive and negative modes using collision-induced dissociation (CID) and data-dependent acquisition (autoMS/MS). High-intensity ions (>5,000 counts) were selected for fragmentation within a 10 Da window and subjected to a 2.5 s acquisition cycle. Fragmentation energy ranged from 10 to 50 eV, adjusted to ion mass and intensity, with a 30 s exclusion window (except for ions with >3 × intensity increase). Mass spectrometry data were processed in MetaboScape 4.0 (Bruker). Recalibration (< 1 ppm, using sodium formate internal standards), peak detection, and retention time correlation (minimum *r* = 0.7) were applied. Only ions present in ≥10% of samples with intensity >5,000 counts were retained. Different charge states (±1, ±2, ±3) and adducts were grouped, and peak areas were integrated to generate a semi-quantitative matrix of metabolites, defined by their neutral mass and retention time. Quality control (QC) and blank samples (every 6 injections and triplicates, respectively) ensured analytical consistency. Initial metabolite annotations were based on ion mass and isotopic pattern using CyanometDB 1.0 (Jones et al., 2021) and NPAtlas 2.0 (van Santen et al., 2022). Additional annotations were performed by comparing MS^2^ fragmentation profiles with GNPS, NIH, MS-DIAL, and EMBL spectral databases (parameters: *m/z* tolerance = 0.02 Da; ≥4 matched peaks; topK = 10; cosine score ≥ 0.70). Metabolomic analyses were performed with MetaboAnalyst 6.0, an online platform for metabolomics data (https://www.metaboanalyst.ca/) using standard parameters and integrated PCA and PLS-DA to produce top metabolites VIP scores plot (Pang et al., 2024). PERMANOVA followed by pairwise tests were performed using euclidean distances.

### 16S rRNA sequencing of bacterial communities and analysis

Following DNA extraction, a PCR1 was performed to amplify a fragment of bacterial 16S rRNA-encoding genes on all samples using primers 341F (5′-CCTACGGGNGGCWGCAG−3′) and 806R (5′-GGACTACVSGGGTATCTAAT−3′) Parada et al., 2016, following standard instructions. The PCR program consisted of an initial 3-min denaturation at 94 °C, followed by 35 cycles with a 45-s denaturation step at 94 °C, 1-min hybridization at 55 °C, and a 1 min 30 s elongation step at 72 °C. Amplification was verified by 1% agarose gel electrophoresis. The PCR1 products were sequenced using Illumina MiSeq 250 × 2 bp at the Genotoul platform (Toulouse, France). Sample accession numbers are summarized in Supplementary Table S2. Amplicon sequence analysis was performed using the QIIME2 pipeline (Bolyen et al., 2019) (version 2022.8). Amplicon Sequence Variants (ASVs) were obtained with the DADA2 algorithm; forward and reverse reads were trimmed at 230 and 220 bp, respectively. The expected error rate was set at 2. Reads with a phred score < 20 and chimeras were discarded. ASVs were then affiliated taxonomically using the SILVA 138.2–99 SSU database (Yilmaz et al., 2014) and chloroplast- and eukaryote-affiliated reads were discarded. Further analyses were done using R studio and the phyloseq package (McMurdie and Holmes, 2013).

## Results

Survival rates were monitored in both species during the 4-month depuration. After 4 months, lower mortality rate was observed in *Lucinoma borealis*, with 13.6% mortality compared to 24.4% in *Loripes orbiculatus*.

### Impact of sulfides starvation on symbiotic load and activity in gills

**Figure 1:**
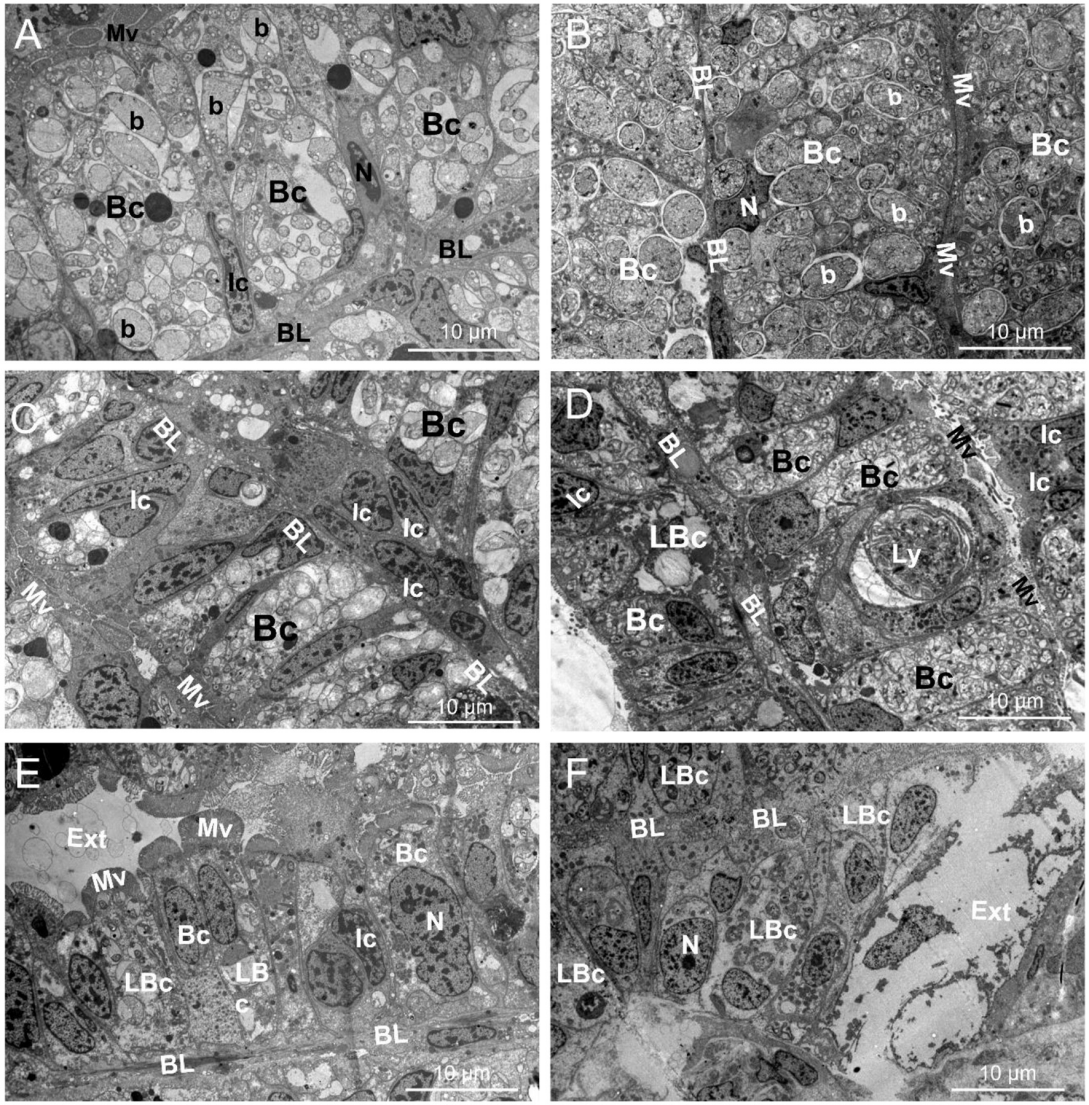
TEM pictures showing the gill ultrastructure (T_0_) and its modifications in *Lucinoma borealis* and *Loripes orbiculatus* during starvation. Gills sections of *Lu. borealis* at time points *T*_0_*, T*_30_, and *T*_120_ days [**(A, C, E)**, respectively]. Gills sections of *Lo. orbiculatus* at time points *T*_0_*, T*_30_, and *T*_120_ days [**(B, D, F)**, respectively]. Each bacteriocyte (Bc) has a nucleus (N) and is oriented from the basal lamina (BL) toward the apical pole, which is characterized by microvilli (Mv). The Bc harbors several symbiotic bacteria (b) within it. Several intercalary cells (Ic) may be observed, as well as lysed Bc (LBc), putative lysosome (Ly), and the external environment (Ext). Images from other specimens are available in Supplementary Figures S1, S2.

At T0, the ultrastructure of *Lu. borealis* and *Lo. orbiculatus* gills displayed inflated bacteriocytes, densely packed with symbiotic bacteria (Figures 1A, B and Supplementary Figures S1, S2). Each bacteriocyte was polarized, with a basal pole anchored to the basal lamina and an apical pole characterized by the presence of microvilli. Nuclei were observed within bacteriocytes or within the sparsely distributed intercalary cells, interspersed between the bacteriocytes. There was no sign of tissue lysis, and both the basal lamina and the microvilli were well visible. After 30 days of starvation, bacteriocytes were comparatively deflated in both species, and symbiont density within these cells decreased. In *Lu. borealis*, intercalary cells appeared to be more numerous amongst the bacteriocytes, and bacterial membranes appeared more wrinkled; however, the overall gill structure kept its well-aligned bacteriocytes. In *Lo. orbiculatus*, a few intercalary cells were also observed, but clear signs of tissue lysis affected both bacteriocytes and the gill epithelium. Symbiont density followed the same decreasing trend as in *Lu. borealis*, with some bacteriocytes completely emptied, significantly disrupting the gill organization. Rare putative lytic structures (lysosomes) were observed only in *Lo. orbiculatus* (Figures 1C, D and Supplementary Figures S1, S2). After 120 days of starvation, signs of tissue lysis were evident in *Lu. borealis* gill sections, where bacteriocytes appeared largely reduced in size compared to T0, with only a few remaining symbionts; however, despite these alterations, the overall gill architecture was relatively preserved. In *Lo. orbiculatus*, severe gill degradation was observed, with most of the gill structure lysed, leaving only a few bacteriocytes still harboring intracellular bacteria, while the majority of the bacteriocytes and the gill epithelium appeared empty and lysed (Figures 1E, F and Supplementary Figures S1, S2).

### Variations in symbiont abundances during starvation

**Figure 2:**
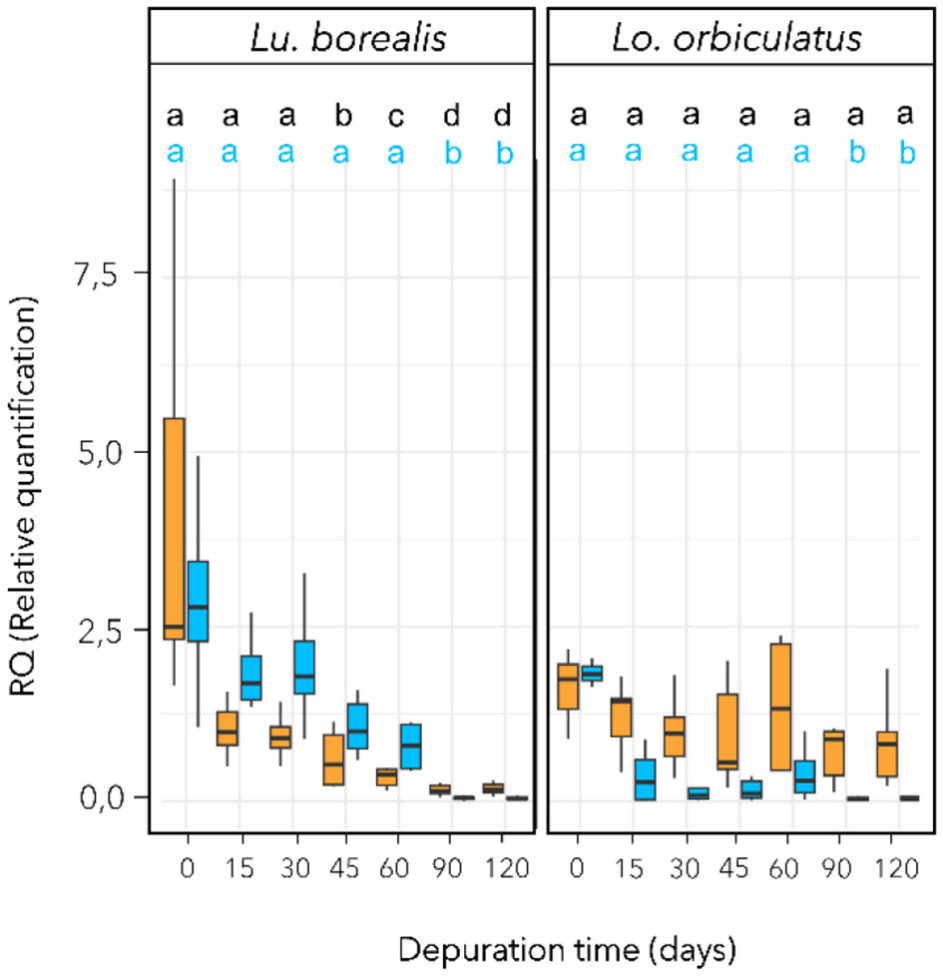
Symbiotic density and activity monitoring during sulfide starvation using qPCR targeting the symbiont 16S rRNA-encoding gene. Symbiotic density variation with time appears in blue (based on extracted DNA) and symbiotic activity in orange (based on cDNA). Different letters refer to groups of significance compared to T_0_. RQ (Relative quantification) is normalized vs. host 18S rRNA-encoding gene copy numbers using the ΔΔCt method. To improve clarity, letters for the cDNA significance level are in black. n = 10 individual per time point. Horizontal bar corresponds to median value, and vertical bar to the data dispersion.

For *Lu. borealis*, statistical analysis revealed a variation in symbiont 16S rRNA encoding gene copy numbers over time (*Kruskal-Wallis*, χ^2^ = 40.6, *df* = 5 *p*_*adj*_ < 0.001, Figure 2). Symbionts 16S rRNA copy numbers (based on cDNA) also decreased over time (*Kruskal-Wallis*, χ^2^ = 38.3, *df* = 5, *p* < 0.001) with a reduction after 60 days (*p*_*adj*_ < 0.001) and a highly reduction after 90 days (*p*_*adj*_ < 0.001) (Figure 2). For *Lo. orbiculatus*, qPCR data revealed a variation in symbiont 16S rRNA encoding gene copy numbers over time (*Kruskal-Wallis*, χ^2^ = 16.1, *df* = 5, *p* < 0.01, Figure 2). *Post hoc* Dunn tests with FDR correction indicated the decrease became significant after 90 days (*p*_*adj*_ < 0.001). Variations in symbiont in 16S rRNA copy numbers (based on cDNA) were not significant (*p*_*adj*_ > 0.05). A PERMANOVA test based on Euclidean distances (Pareto-scaled data) showed that the DNA and cDNA datasets are strongly structured by time, species, and their interaction (overall *R*^2^ ≈ 0.58, *p* < 0.001). The time × species interaction confirms that temporal trajectories differ between species. Taken together, these results further support an effect of time and species on symbiont dynamics, characterized by a progressive decrease in load, and, to a lesser extent, loss of activity, with species-specific kinetics.

### Bacterial community composition in the gills and visceral mass

**Figure 3:**
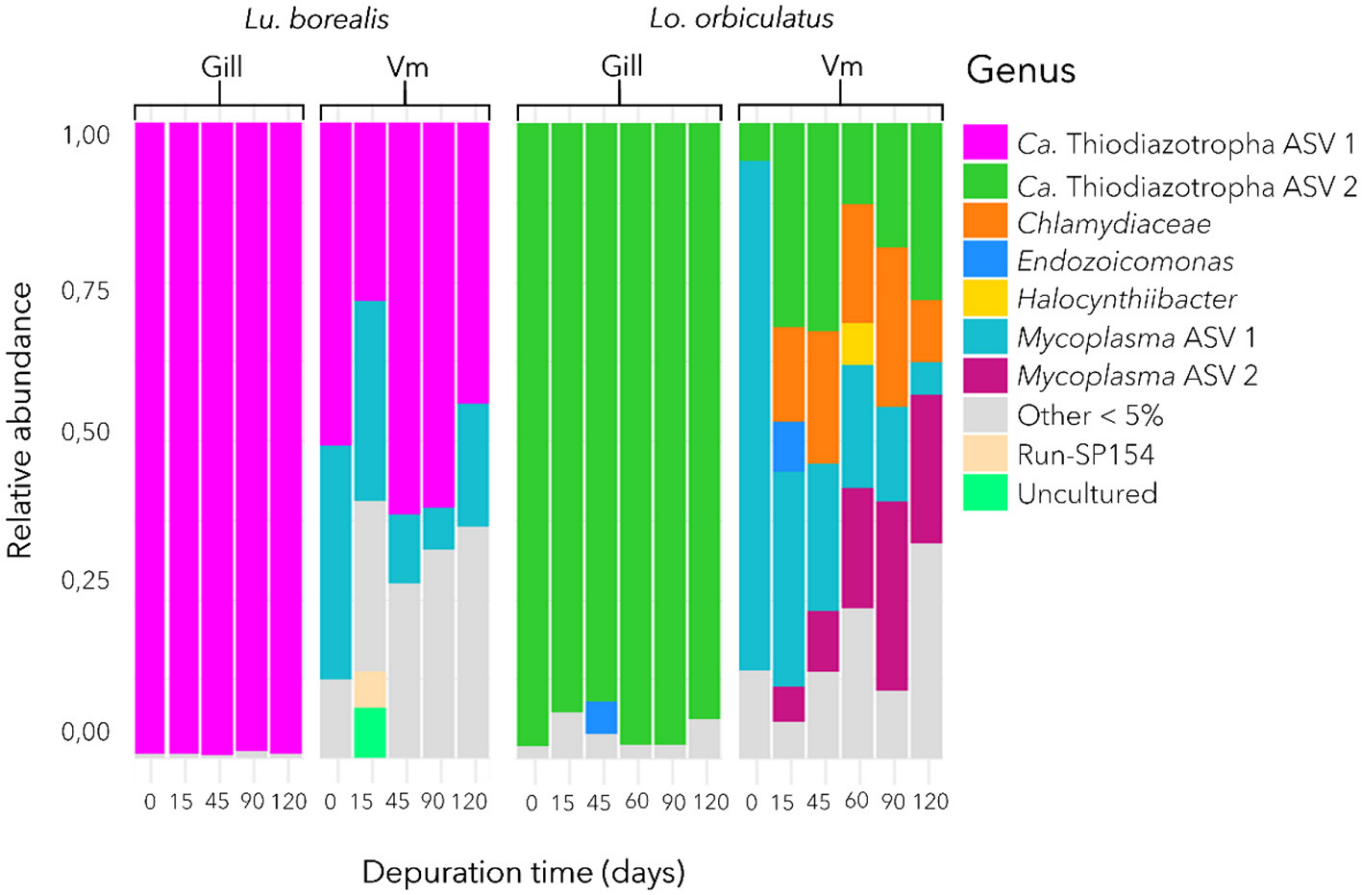
Bacterial community composition in the gill and visceral mass (Vm) of *Lu. borealis* and *Lo. orbiculatus* during starvation. Average relative abundance of bacterial taxa from 6 specimens per time point. *Ca*. Thiodiazotropha endolucinoma ASV1 are displayed in purple, while *Ca*. Thiodiazotropha endoloripes ASV2 are shown in green.

In each of the two species, a single dominant ASV was identified in the gills, corresponding to *Ca*. Thiodiazotropha (Figure 3). The one found in *Lu. borealis* matched *Ca*. Thiodiazotropha endolucinoma, previously identified in *Lucinoma borealis* (GenBank accession number LT548924.1; nucleotide identity = 100%), while the ASV found in *Lo. orbiculatus* matched *Ca*. Thiodiazotropha lucinalis found in *Loripes orbiculatus* (GenBank accession number LT548933.1; nucleotide identity = 100%). These two ASVs differ by nine base pairs. Bacterial communities from the visceral mass also displayed abundant *Ca*. Thiodiazotropha ASVs, as well as representatives of other phyla (especially *Mycoplasma* sp. found in both species and *Chlamydiaceae* sp. found only in *Lo. orbiculatus*). In *Lo. orbiculatus*, ASVs belonging to the *Endozoicomonas* genus were found in the gills only at 45 days of starvation, and only after 15 days of starvation in the visceral mass.

### Ultrastructure and morphotypes of symbionts

**Figure 4:**
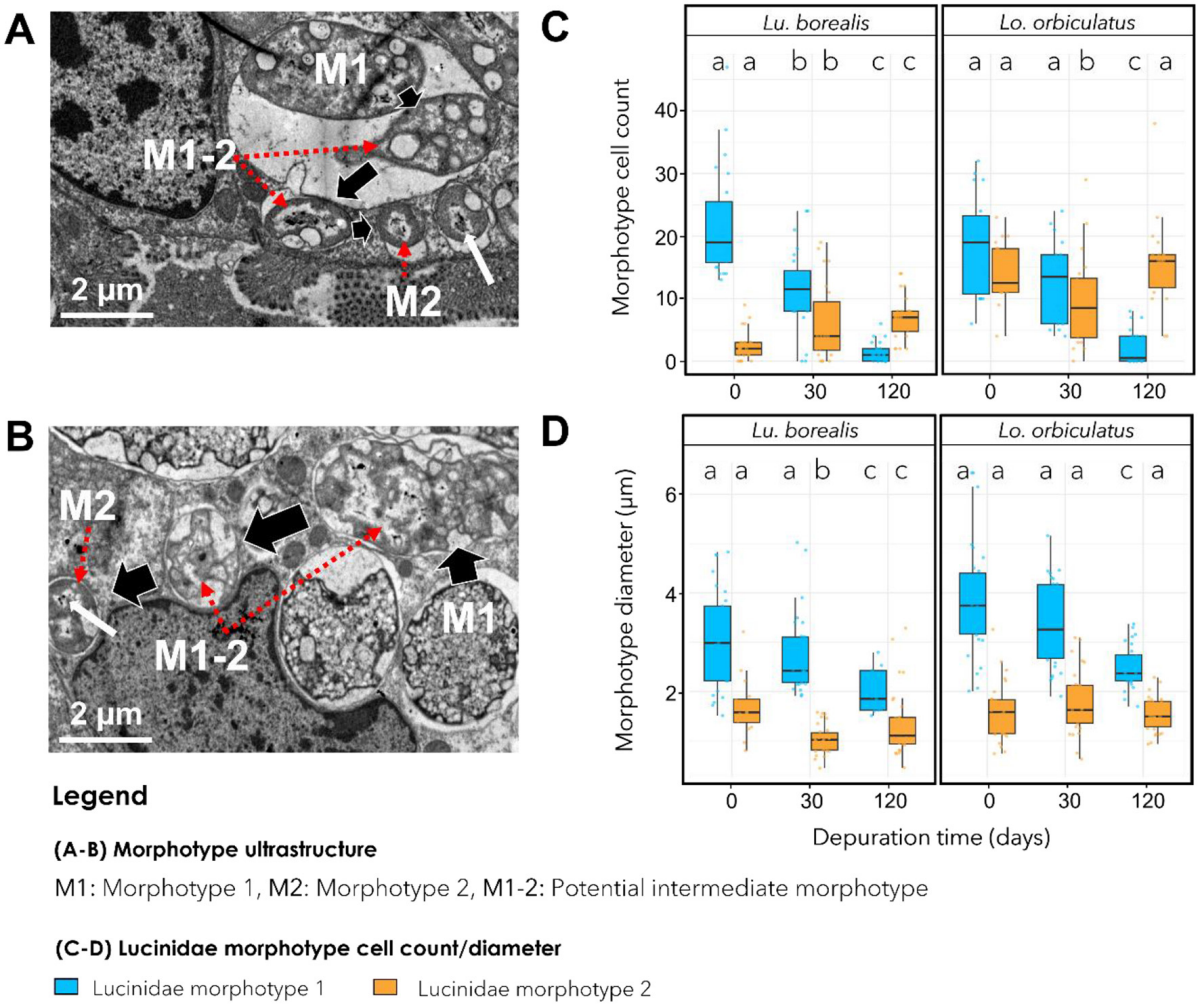
Transmission electron microscopy (TEM) images showing symbiont morphotypes. **(A)** Gills of *Lu. borealis* at T0. **(B)** Gills of *Lo. orbiculatus* at T0. Two morphotypes are visible, labeled M1 and M2, and an intermediate morphotype is labeled M1-2. Red arrows indicate M1-2 and M2 symbiont morphotypes. Key characteristics of M2 are cell diameter and the presence of potential condensed DNA within the cytoplasm, indicated by a white arrow. **(C)** Average number of cells displaying each morphotype in a bacteriocyte during starvation. **(D)** Variation in morphotype diameter in both species throughout starvation. Colors indicate morphotype 1, blue; morphotype 2, orange. Different letters indicate statistically significant differences in morphotype number or size (Kruskal-Wallis test followed by Dunn's *post hoc* test, with *p*-values adjusted using the FDR method). Errors bars indicate data dispersion.

TEM images revealed distinct bacterial morphotypes in the gills of each species. In *Lu. borealis*, symbionts appeared large (mean T0 diameter = 3.07 ± 1.00 μm, *n* = 40), displaying diverse shapes, with few vacuoles and no electron-dense granules (Figure 4A). In contrast, in *Lo. orbiculatus*, symbionts seemed larger (mean T0 diameter = 3.87 ± 1.15 μm), consistently round-shaped, and contained numerous electron-dense granules (Figure 4B). In both species and at all time points, two clearly distinct morphotypes were visible, hereafter labeled as morphotype 1 (M1) and morphotype 2 (M2). M1 corresponds to the most abundant and M2 to the least abundant morphotype. In both species, M2 cells were consistently smaller than M1 (M2 mean T0 diameter = 1.68 ± 0.58 μm for *Lu. borealis* and 1.59 ± 0.57 μm for *Lo. orbiculatus*) and appeared as ovococcus-shaped bacteria (Figures 4A, B). During starvation, the number of M1 morphotypes per bacteriocyte gradually decreased over time, and after 120 days, only a few remained (Figure 4C). Conversely, the M2 numbers slightly increased (Figure 4C). The diameter of M1 cells decreased over time, whereas that of M2 cells remained stable (Figure 4D).

Morphotypes displaying intermediate features between M1 and M2 were observed, particularly in *Lo. orbiculatus*, characterized by smaller diameter compared with M1, membrane modification, and the appearance of putative condensed DNA reminiscent of that visible in M2 (Figures 4A, B). Overall, TEM imaging indicated a decrease in symbiont density [mostly of the largest morphotype, M1, in both species over time (Figures 1A–F)].

### Metabolome composition in gills and visceral mass

**Figure 5:**
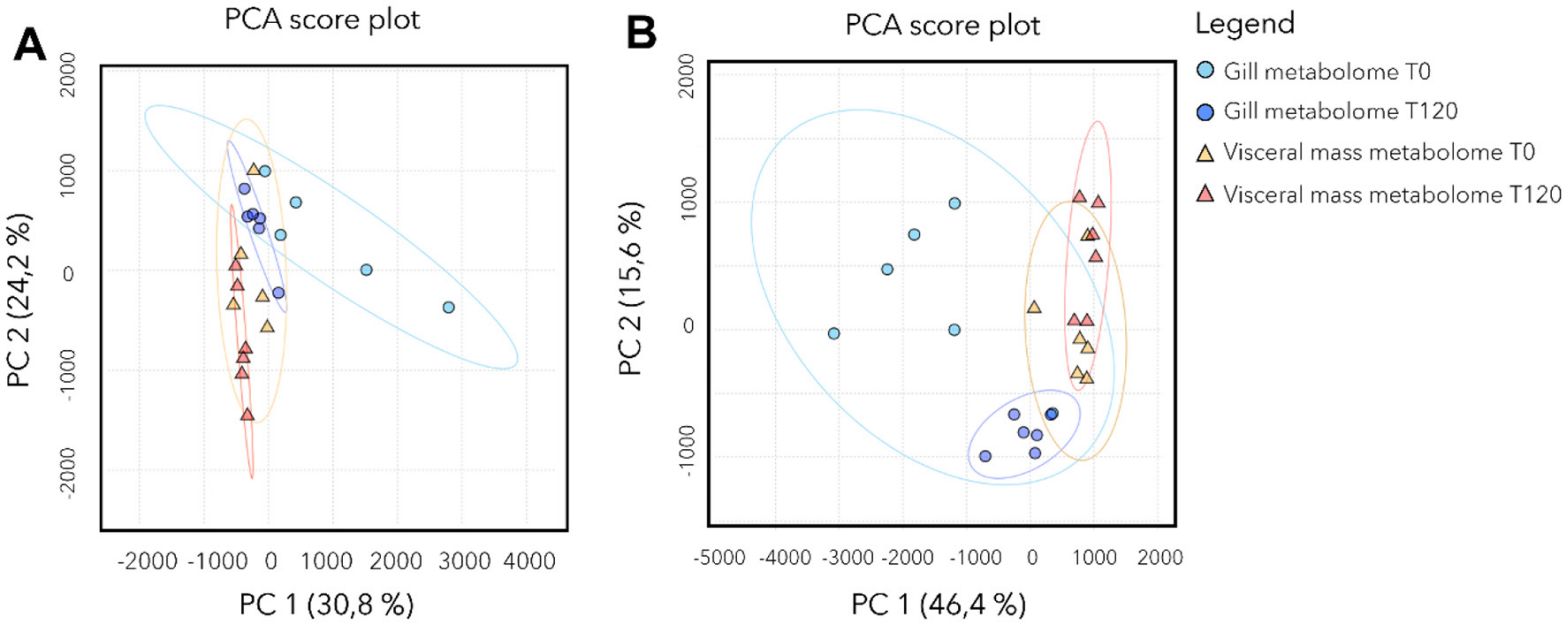
Metabolome composition of *Lu. borealis* and *Lo. orbiculatus* tissues (gills and visceral mass) during starvation. PCA of the metabolomic profiles in the *Lu. borealis* **(A)** and *Lo. orbiculatus* **(B)** gills and visceral mass at T0 and T120 days of starvation.

A total of 3,317 features were detected, of which 321 (~10%) were annotated. PCA showed a clear separation between species and, within species, differences between gill and visceral mass (Supplementary Figure S3). For *Lucinoma borealis* and *Loripes orbiculatus*, PERMDISP (respectively global ANOVA *p* ≈ 0.44 and *p* ≈ 0.22), indicating comparable dispersions across groups. For *Lu. borealis*, a two-way PERMANOVA (Euclidean, 999 permutations) run on T0 and T120 days of sulfide starvation revealed effects of tissue and time, as well as a tissue × time interaction (tissue: *p* = 0.001; time: *p* = 0.003; tissue × time: *p* = 0.037). *Post-hoc* pairwise tests (adonis, FDR) supported a temporal shift in gills between T0 and T120 (*p* < 0.01, *p*_*adj*_ ≈ 0.03), tissue differences at both time points (gill T0 vs. visceral mass T0 *p*_*adj*_ ≈ 0.018; gill T120 vs. visceral mass T120: *p*_*adj*_ ≈ 0.03), while the visceral mass T0 vs. T120 contrast was not significant after correction (*p*_*adj*_ ≈ 0.16). PCA consistently shows time-related changes in gills from T0 to T120, superimposed over a strong separation by tissue type. For *Lo. orbiculatus*, PERMANOVA (Euclidean, 999 permutations) showed a strong tissue effect, a marginal time effect, and a tissue × time interaction (tissue: *p* = 0.001; time: *p* ≈ 0.053; tissue × time: *p* ≈ 0.029). Pairwise tests confirmed tissue differences at both time points (gill T0 vs. visceral mass T0 and gill T120 vs. visceral mass T120, *p*_*adj*_ ≤ 0.045), whereas within-tissue temporal contrasts (gill T0 vs. gill T120; visceral mass T0 vs. visceral mass T120) were not significant after FDR (e.g. *p*_*adj*_ ≈ 0.13–0.26). In both bivalves, tissue is the primary driver of metabolome structure. A time effect is also detected in both species, but it is clearer in *Lu. borealis* gills (significant shift from T0 to T120) than in *Lo. orbiculatus* (where time effects are modest once tissues are considered). In both species, the PCA (Figure 5) show two clear trends: the loss of metabolome diversity, and a shift of the gill metabolome composition at T120 that drives it closer to that of the visceral mass.

Heatmap of the annotated metabolites shows that the two species have a specific gill metabolome. We didn't detect peptides in *Lu. borealis* contrary to *Lo. orbiculatus*. ANOVA tests followed by *post hoc* Tukey tests (*p*-value adjusted using the FDR method with a threshold of 0.05) identified the 20 most discriminant annotated metabolites (Supplementary Figure S4). For *Lu. borealis* gills, the main dysregulated metabolites were lipids including phosphoethanolamines (PE) and lysophosphatidylcholine (LPC), ceramides, singleton amino acids, saccharides, nucleic acids and glutathiones. All these compounds decreased in abundance with time. Regarding the visceral mass of *Lu. borealis*, we found only 9 metabolites that displayed changes. Some metabolites concentration increased with time (ceramides and LPC), while others decreased (oleoylethanolamine, adenosine, uridine, together with other nucleic acids). For *Lo. orbiculatus* gills, Two PE emerged as the most statistically significant features (FDR = 5.25e^−09^). These lipids exhibited high abundances at T0 followed by a pronounced decline at T120. In parallel, multiple peptides demonstrated elevated abundance at T0, followed by a decrease at T120. Notably, despite the presence of subtle shifts in several nucleotides and small peptides, the overall structure of the heatmap reveals clear temporal clustering, with T0 samples exhibiting relatively similar metabolomic signatures, and T120 samples forming a distinct group (Supplementary Figure S4). For *Lo. orbiculatus* visceral mass, heatmap reveals clear temporal trends in the abundance of several key metabolites. Notably, PE showed significantly higher abundances at T0 compared to T120. In contrast, saccharides and nucleosides remained relatively stable across all time points. Interestingly, several ceramides and sphingolipids became more abundant at T120. Collectively, these patterns highlight distinct classes of metabolites exhibiting time-specific responses, with lipid remodeling and ceramide accumulation emerging as key signatures of late-phase metabolic acclimatization in *Lo. orbiculatus* tissues. Overall, the two species thus exhibit contrasting tissue-specific metabolic responses over time.

### Response of apoptosis-related genes during starvation

**Figure 6:**
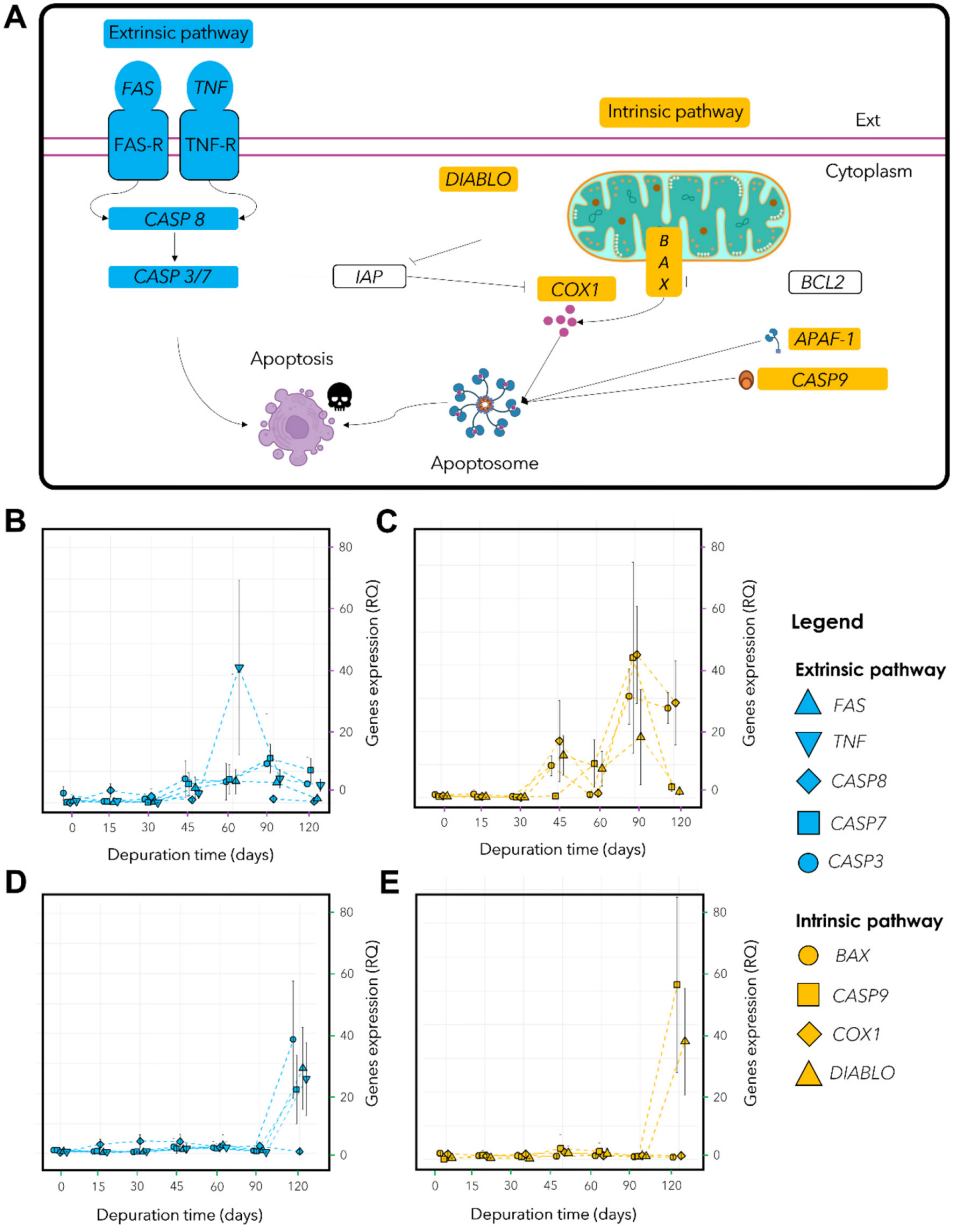
Host apoptotic response in *Lu. borealis* and *Lo. orbiculatus* monitored by qPCR. **(A)** Schematic view of targeted genes involved in eucaryote apoptotic pathways. Extrinsic pro-apoptotic genes are labeled in blue, intrinsic pro-apoptotic genes in yellow, and main anti-apoptotic genes are represented in white. **(B)** Relative quantification (RQ) of genes involved in extrinsic apoptotic pathway *(FAS, TNF, CASP8, CASP7, CASP3)* in *Lu. borealis* and **(D)** *Lo. orbiculatus*. **(C)** Relative quantification (RQ) of genes expression involved in intrinsic apoptotic pathway *(BAX, DIABLO, COX1, CASP9)* in *Lu. borealis* and **(E)** *Lo. orbiculatus*. **(B–D)** Right axis corresponds to the relative quantification (RQ) of apoptotic gene expression normalized vs. host 18S rRNA-encoding gene using the ΔΔCt method. n = 10 specimens per time point.

For *Lu. borealis*, expression levels of apoptosis-related genes involved in the extrinsic pathway increased from day 45 onwards, with a marked overexpression peak of *TNF* at 60 days (Figures 6A, B). *FAS* was upregulated at 45 days (*p*_*adj*_ < 0.001), *TNF* at 60 days (*p*_*adj*_ < 0.01), *CASP8* at 15 days (*p*_*adj*_ < 0.001), and *CASP7* at 60 days (*p*_*adj*_ < 0.05), *CASP3* expression did not vary significantly (*p*_*adj*_ > 0.05). For *Lu. borealis* intrinsic pathway, two peaks of overexpression were observed at 45 and 90 days, separated by a transient decrease between 45 and 60 days (except for *CASP9*), and followed by a decline after 90 days (Figure 6C). Non-parametric Kruskal-Wallis tests followed by *post hoc* Dunn's tests (Benjamini-Hochberg correction) revealed overexpression for *BAX* and *COX1* at 90 days (*BAX*: *p*_*adj*_ < 0.001; *COX1*: *p*_*adj*_ < 0.001), *DIABLO* at 45 days (*p*_*adj*_ < 0.001), *CASP9* at 60 days (*p*_*adj*_ < 0.01), and *CASP7* at 45 days (*p*_*adj*_ < 0.05). As for the extrinsic pathway, *CASP3* expression did not vary significantly.

Among the genes involved in the extrinsic pathway found in *Lo. orbiculatus, CASP8* was overexpressed as early as day 15 (*p*_*adj*_ < 0.001), while *FAS* (*p*_*adj*_ < 0.01), *TNF* (*p*_*adj*_ < 0.01), and *CASP7* (*p*_*adj*_ < 0.05) showed increase after 60 days. *CASP3* expression remained low and stable (Figure 6D). The expression of genes involved in the intrinsic pathway remained low and stable until 90 days, followed by a sharp upregulation at 120 days for *DIABLO* (*p*_*adj*_ < 0.001), *CASP7* (*p*_*adj*_ < 0.01), and *CASP3* (*p*_*adj*_ < 0.01). *BAX* and *COX1* did not show significant changes in expression. *CASP9* was upregulated from day 15 (*p*_*adj*_ < 0.01) (Figure 6E). These findings indicate different responses between the two lucinid species, reflecting distinct apoptotic regulation strategies in response to sulfide starvation and symbiont depletion.

## Discussion

### *Lucinoma borealis* and *Loripes orbiculatus* survive a 4-months sulfide starvation despite decrease in symbiont abundances

*Lu. borealis* and *Lo. orbiculatus* both displayed limited mortality over 4 months, showing resistance to prolonged starvation despite a slow and progressive decrease in gill symbiont abundances. The remaining symbiotic bacteria were still metabolically active despite the absence of hydrogen sulfide (H_2_S), their preferred substrate. These results are consistent with previous findings on the tropical species *Codakia orbicularis* (Codakiinae), which inhabits mangrove environments. In this species, it was shown that the chemoautotrophic symbiont population—initially occupying 32.4% of the gill tissue volume—dropped to only 1.7% after 3 months of depuration, although the remaining symbionts remained metabolically active (Caro et al., 2009). However, in another lucinid (*Lucina pensylvanica*), no decrease in symbiont abundance was observed after 6 months of starvation, but elemental sulfur content tended to zero after 3 months of starvation, illustrating that endosymbiotic population regulation is strongly host-species-dependent in lucinids (Elisabeth et al., 2014). In the present study, qPCR and TEM data both indicate a progressive decrease in symbiont densities in the absence of sulfide in both *Lu. borealis* and *Lo. orbiculatus*. *Lu. borealis* and *C. orbicularis* both belong to the subfamily Codakiinae, suggesting that this trait may be shared within this group. However, *Lo. orbiculatus* belongs to another, the Lucininae (Taylor et al., 2022), and the observation of a similar decrease may point to a common adaptive strategy among coastal Lucinidae. Similar responses in some tropical and temperate coastal species can thus be assumed. However, these responses may not be valid for all lucinids, as exemplified by the results on *Lucina pensylvanica*, and because the family colonizes diverse habitats including coastal area sediments, seagrass meadows, mangroves, or deep-sea ecosystems like deep shelf and cold seeps (Duperron et al., 2013; Osvatic et al., 2023). Members of the Codakiinae subfamily, and the *Lucinoma* genus with the species *Lucinoma kazani*, are frequently found associated with deep-sea cold seeps (Salas and Woodside, 2002; Duperron et al., 2007), and would be interesting target species to compare the physiological responses between coastal and deep-sea *Lucinoma* to sulfide starvation.

**Figure 7:**
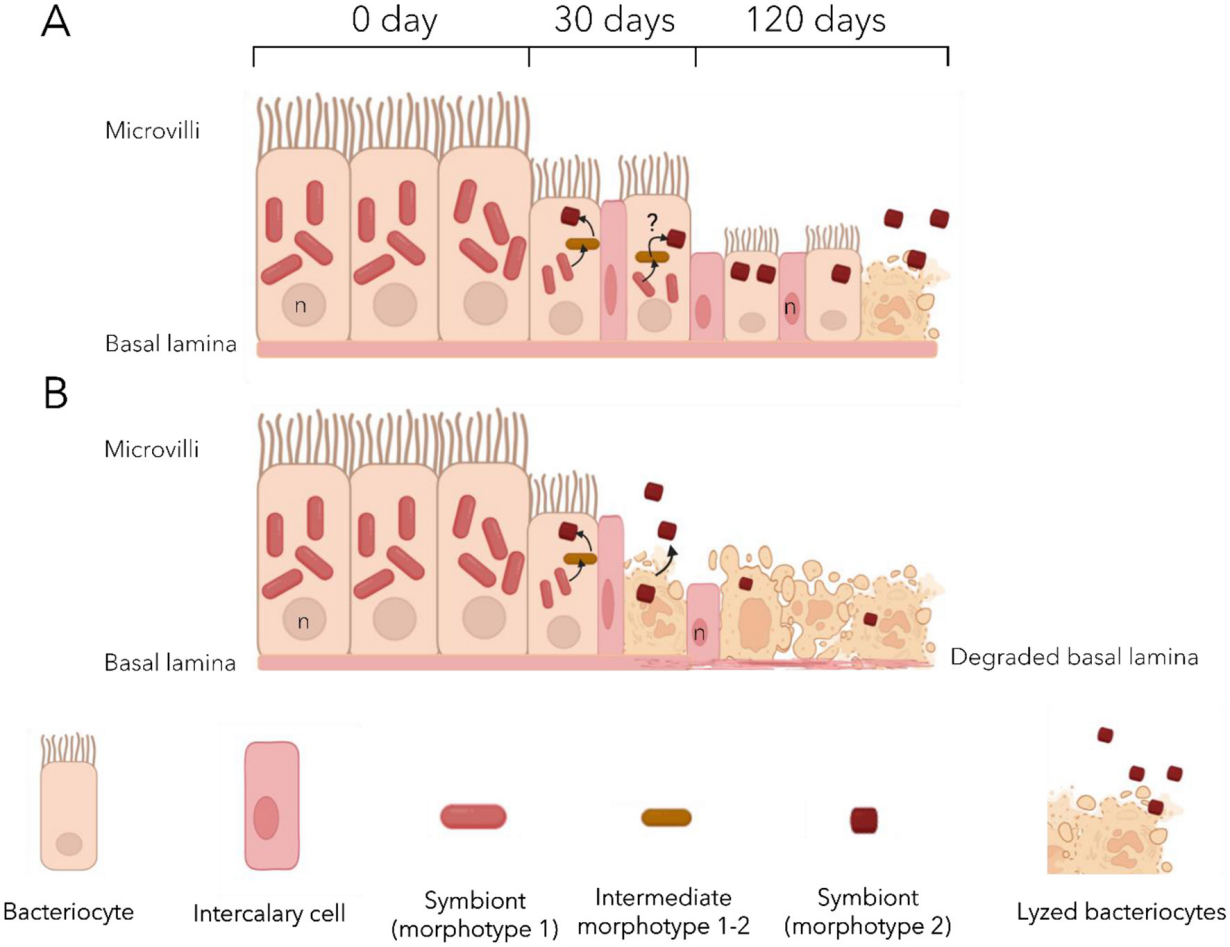
Proposed model of the effects of sulfide deprivation on the gill epithelium of *Lucinoma borealis* **(A)** and *Loripes orbiculatus* **(B)**. Initially, the gill epithelium displays a well-organized structure, composed mainly of large bacteriocytes densely packed with symbionts, mostly morphotype 1 cells. After 30 days bacteriocytes shrink with a marked reduction in morphotype 1 symbionts. Morphotype 1–2, may occasionally be observed, hypothetical transitions between morphotypes are indicated by black arrows. Other cell types (intercalary and goblet cells) are observed. After 120 days, many bacteriocytes undergo lysis, releasing symbionts into the environment. Remaining bacteriocytes are reduced in size and contain at most a few morphotype 2 cells, while morphotype 1 is almost absent. Epithelial disorganization occurs earlier in *Lo. orbiculatus* with lysed bacteriocytes already observed after 30 days.

Under maintenance conditions used in the present study, both species appeared to maintain a good physiological state, as no sign of infection or dysbiosis was detected. Indeed, symbionts remained the main bacteria in the gills up to the end of the experiments, and bacterial communities did not experience major changes in the visceral mass either. Metabolomic analyses revealed that each species exhibits its own profile, both in the gills and in the visceral mass. During starvation, the gill metabolome appeared to increasingly resemble that of the non-symbiotic tissue over time in both *Lu. borealis* and *Lo. orbiculatus*. This convergence likely results from symbiont loss, leading to the decreased abundance of symbiont-derived metabolites. Furthermore, metabolomic analysis highlighted changes in the metabolite content of both the gills and the visceral mass in both species over time. These metabolome modifications are proxies for functional changes within the tissues, indicating functional alterations. Yet, the hosts were able to survive throughout the depuration process, suggesting acclimatization to sulfide deprivation and symbiont loss. Gill tissue architecture was notably altered in both species during starvation, suggesting a response to symbiont loss and nutritional stress (Figure 7). Because no sign of dysbiosis—such as the emergence or overrepresentation of environmental bacterial genera—was detected, and no potentially pathogenic genera were identified through metabarcoding, we assume that tissue modification does not result from infection by opportunistic pathogens or a shift of symbionts from mutualists to exploitative phenotypes under host stress and reduced control.

The observation of two distinct bacterial morphotypes within the bacteriocytes of both lucinids—both of them likely belonging to the same ASV according to the metabarcoding results—suggests another type of symbiont plasticity in response to the lack of sulfide. It may involve a morphological change from a large active form, which abundance decreases with time, to a smaller less active form which abundance remains more stable (Figure 7). Coupled with the progressive loss of symbionts, this strongly suggests that gill tissue alteration is unlikely to result from host physiological collapse or microbial infection. We suggest that the tissue alteration could be the consequence of an apoptotic response triggered by the host in order to replace useless empty bacteriocytes and non-functional cells, so as to recycle cellular components. We also observed an increasing number of undifferentiated cells—the intercalary cells—in the lateral zone of the gill filaments after 30 days of starvation, when the bacteriocytes began to deflate. In *C. orbiculata*, long-term starvation led to modifications in cell organization in the lateral zone of gill filaments in response to variation in the number of bacteriocytes and granule cells, and host tissue regeneration in gill filaments may occur by both replication of existing cells and division of undifferentiated cells (Elisabeth et al., 2012). Thus, we suggest that the same process occurs in both Lucinidae from this study, maintaining the integrity of the gill epithelium.

### Differences in functional response to sulfide starvation between *Lu. borealis* and *Lo. orbiculatus*

Aside from the aforementioned similar trends, differences were also observed. First of all, each species was consistently associated with its own symbiont 16S rRNA phylotype, despite the co-occurrence of *Lu. borealis* and *Lo. orbiculatus* in seagrass beds, emphasizing strong host–symbiont fidelity. Host responses to sulfide starvation also displayed differences. First, *Lo. orbiculatus* displayed relatively higher mortality rates compared to *Lu. borealis*. Yet, the most striking differences were related to the activation of apoptotic response. The latter is intriguing given the ubiquity and high conservation of apoptosis pathways across eukaryotes (Koonin and Aravind, 2002; Kaushal et al., 2023), and even more so among two species belonging to the same family (Quistad et al., 2014). Apoptosis may be initiated by the host to recycle non-functional cells, such as empty bacteriocytes or cells affected by oxidative stress, in order to mitigate damage and recover nutrients via autophagy (Piquet et al., 2019) as well as re-organize tissues (Gros et al., 2012). In *Lu. borealis*, the intrinsic pathway response followed a well-defined temporal trajectory, indicative of a structured and host-regulated apoptotic process, including an alternation of pro-apoptotic phases followed by a down regulation of the apoptotic response. Pro-apoptotic phases consisting in the expression of *BAX* and *DIABLO* recruiting caspase 9 and, with *COX1* and *APAF*, forming the apoptosome pathway (Mai et al., 2023). Such a temporally modulated apoptotic response suggests a host-regulated physiological mechanism, indicating that the bivalves remained in good condition throughout the experiment. However, gene expression data alone is not sufficient to demonstrate the role of apoptosis as a regulating mechanism of the symbiosis. Alternatively, apoptosis could help getting rid of oxidatively damaged host cells (Hinzke et al., 2019; Piquet et al., 2019) or be involved in tissue reorganization regardless of its symbiotic status. This has been shown for example in the rapid turn-over of the ciliated cells (the first cells in contact of surrounding fluids and toxics) and hemocytes (putative role in detoxification) of *Bathymodiolus azoricus* and *Bathymodiolus puteoserpentis* (Piquet et al., 2019) as well as in the shallow water Lucinidae *Codakia orbiculata* (Gros et al., 2012). *Lo. orbiculatus* on the other hand displayed a different pattern of apoptosis gene regulation. Indeed, results suggest either no structured response, or at least a response occurring very late, at the last time point (120 days). This could either reveal a very delayed apoptosis induction, or uncoordinated cellular response to stress. In the latter case, the inability to activate controlled apoptosis could represent a strategy to preserve gills tissue integrity and maintain remaining symbionts. These results suggest that lysis in tissues observed by TEM is not the consequence of an apoptotic response but rather a possible necrosis, which has to be tested.

Besides the apoptotic response, some annotated metabolites of which abundances varied during sulfide deprivation differed between *Lu. borealis* and *Lo. orbiculatus*. In *Lu. borealis*, the gill response appeared more dynamic compared to the gills of *Lo. orbiculatus*. Lipids such as LPC and PE, along with ceramides, increased over time. These lipids may be related to symbiont membranes, but their accumulation does not seem congruent with the symbiont loss observed after 120 days of starvation. Membranous lipids like LPC and PE play diverse biological roles, ranging from structural membrane components to signaling molecules; however, their functions remain poorly understood in marine invertebrates. The marked increase in ceramide levels observed in the gills of *Lu. borealis* after 120 days may on the other hand be associated with the activation of apoptosis. Indeed, ceramides are known to participate in stress responses in marine mollusks, immune function (Timmins-Schiffman and Roberts, 2012), and the initiation of apoptosis via intrinsic signaling pathways (Jain et al., 2017; Dadsena et al., 2019). The elevated ceramide levels are consistent with the observed apoptotic response. In the visceral mass of *Lu. borealis*, in contrast to the gills, all the most discriminant metabolites, such as lipids and glutathione, decreased over time. These changes may reflect a coordinated response involving oxidative stress management via glutathione balance (Cnubben et al., 2001; Gasmi et al., 2024) and membrane remodeling, potentially involving ceramides. The reduction in the reduced form of glutathione suggests a shift toward its oxidized form, indicating possible increase of oxidative stress in the visceral mass.

The gill metabolome of *Lo. orbiculatus* appears more affected by starvation, with a decrease in nearly all discriminant metabolites. In addition to the reduction in lipids (LPC and PE), all peptides detected in clams at the beginning of the exposure disappeared after 120 days. These peptides may have been associated with symbionts, and their disappearance could be explained by the progressive loss of symbiotic bacteria and the tissue reduction. No ceramide production was detected in the gills, congruent with the observed lack of a coordinated apoptotic response, and suggesting that the observed overexpression of apoptosis-related genes after 120 days may rather correspond to transcriptional dysregulation. The visceral mass of *Lo. orbiculatus* also showed an increase in oxidized glutathione, indicating potential oxidative stress in this tissue as observed in *Lu. borealis*. Differences in lipid profiles (LPC, PE, and ceramides) over time between the two species support the distinct membrane alterations observed by TEM. Indeed, *Lu. borealis*, which retained higher lipid levels after 120 days of depuration, maintained well-organized gill structures, whereas *Lo. orbiculatus* exhibited lysed tissues congruent with a reduced presence of membranous lipids (Figure 7).

Differences between the two species suggest distinct stress response strategies. Given the extreme environmental conditions these bivalves typically inhabit (anoxic, sulfidic sediments), these results emphasize the importance of metabolic adaptation mechanisms. The ability of the gills or visceral mass to manage chronic stress and sustain a functional symbiosis is likely a key determinant of species survival and distribution. Moreover, due to their central role in gas exchange and symbiont housing, gills may represent the most vulnerable and reactive tissue to environmental stressors. Some of the difference in responsive metabolites and metabolic profiles could of course also result from the presence of distinct symbiotic bacteria in the gills of *Lu. borealis* and *Lo. orbiculatus*, and distinct communities in their respective visceral mass, possibly displaying different metabolites composition.

### Holobiont fidelity and plasticity: a recipe to preserve the symbiotic association during sulfide-depleted periods?

In deep-sea chemosymbiotic bivalves, namely hydrothermal vent mussels *Bathymodiolus azoricus* and *Bathymodiolus puteoserpentis*, rapid loss of symbionts and elevated levels of apoptosis affecting the gill bacteriocytes with low symbiont abundances has been observed within a few days under sulfide-depleted conditions (Piquet et al., 2019). These findings underscore the direct influence of environmental conditions on holobionts response to physicochemical stress, particularly with respect to the availability of substrates essential for chemosynthetic bacteria. Once the symbionts disappear, the gill bacteriocytes enter apoptosis. Hydrothermal vents are characterized by frequent and abrupt physicochemical fluctuations associated with the highly dynamic hydrothermal fluid/seawater interface, with important temperature and sulfide levels variations (Le Bris et al., 2006; Lelièvre et al., 2017). Temperate coastal sediments on the other hand tend to experience longer-term, more periodic fluctuations (e.g., tidal cycles, seasonal variations in temperature, eutrophication levels and anthropogenic influences…; Diaz and Rosenberg, 2008; Cloern et al., 2016). These environmental dynamics posit different challenges to chemosymbiotic organisms in order to cope with temporal variability. In this study, several results point to possible adaptations in lucinids and their symbionts. First, despite a decrease, symbionts remained present and active throughout the 4 months. Second, despite that *Lu. borealis* and *Lo. orbiculatus* each harbor a specific symbiont, two distinct bacterial morphotypes were observed in the gills of each host species. We propose that they both represent distinct physiological states of the same symbiont, *Candidatus* Thiodiazotropha sp., with the larger morphotype 1 (M1) representing the metabolically active form, of which abundances and diameter decrease with starvation duration, and the smaller morphotype 2 (M2) representing a dormant or storage form. This hypothesis is supported by the persistent detection of only the symbiont-specific ASV in gills tissues throughout the entire depuration period, by the occurrence of putative intermediate morphotypes between M1 and M2 from time point T0, supported by the progressive reduction in M1 cell diameter over time, and by the predominance of M2 following 30 days of stress—forms that are rarely observed under natural conditions especially in *Lu. borealis*.

On the hosts side, apoptosis in lucinids appears delayed compared to deep-sea hydrothermal vent *Bathymodiolus* mussels. Maintaining low number of less-active symbionts in bacteriocytes during periods of low sulfide availability (lower temperatures, lower activity of the seagrass bed) could be a way to preserve the ability to restore the symbiosis when conditions become favorable again. We thus hypothesize that the holobionts response of coastal Lucinidae, involving fewer active forms of the symbionts and delayed apoptosis, could be adaptations to the temperate seagrass bed habitat. In order to validate this hypothesis, re-exposure to sulfide need to be performed, to test whether dormant M2 cells may revert to the active M1 form.

## Conclusion

*Lu. borealis* and *Lo. orbiculatus* survive and maintain their symbiotic association throughout a 4 months sulfide deprivation, showing great resistance to low sulfide levels. Shared responses include maintenance of host-specific symbionts at low abundance and activity levels, good physiological state, and limited mortality despite altered gills tissue structure. Extrinsic and intrinsic apoptosis occurs very late in both species in contrast to hydrothermal vent mussels, with *Lo. orbiculatus* displaying the latest response. The morphology of gill-associated symbionts changes upon starvation. Overall, observed responses are congruent with a strategy allowing the preservation and maintenance of the association in the absence of sulfide, as can happen during low temperature or low primary production periods in temperate seagrass beds. Delayed apoptosis and maintenance of a small population of symbionts in the gills may be relevant strategies to cope with extended low-sulfide periods that occur in temperate seagrass beds possibly contributing to the ecological success of lucinids in seagrass beds. Co-occurrence as well as relatively easy access and maintenance of *Lu. borealis* and *Lo. orbiculatus* make them suitable model species for the study of coastal chemosynthesis. Further investigation should clarify the role of apoptosis in symbiosis regulation, document the nutritional shift that allows holobionts survival, and evaluate the reversibility of observed changes when sulfide becomes available again.

## Data Availability

The data presented in this study are publicly available. This data can be found here: https://www.ncbi.nlm.nih.gov/sra, accession PRJNA1266712.
