# Supplementary material for: Symbiont retention and holobiont response under simulated sulfide deprivation in Lucinid clams from seagrass beds
Source: Front Microbiol. 2025 Nov 18;16:1637201. doi: 10.3389/fmicb.2025.1637201 (PMC12746666; doi:10.3389/fmicb.2025.1637201)
Supplement: Supplementary file 1 [file Supplementary_file_1.docx]

**Supplementary data**

**Supplementary_table_S1**

| **Species** | **Target gene** | **Forward (5’-3’)** | **Reverse (5’-3’)** |
| --- | --- | --- | --- |
| *Lu. borealis + Lo. orbiculatus* | 18S rRNA | CGCCGGCGACGTATCTTTCAA | CTGATTCCCCGTTACCCGTTAC |
| *Ca.* Thiodiazotropha *sp.* | 16S rRNA | CAGCAGTGGGGAATATTGGAC | AAAGTTAACGTCAAGACCCAAGGT |
| *Lu. borealis* | BAX | ATCGGCAGTTCTCCCAATCC | TCGACCATCTTCTCCATGGC |
|  | Bcl2-1 | GAAAGAAGCGGGATGGGGAA | TCAAGCTGTTCTGGCACTGT |
|  | Bcl2-2 | TCCGACCAGAGTGCAGTAGA | GATTGTGTTGTTACCGCGCC |
|  | Bcl2-3 | GACTTGGCCAGCTCTTTTGC | CCAGTGGTTGCTATGAGGCT |
|  | Caspase 3 | GTGGAAAGGCGGGTTACAGA | TGCCATTTATGTGCGTGCTG |
|  | Caspase 7 | CAGTGAGCGAGGGTGAAGTT | CTCTGAAGCCCCTGGTGAAG |
|  | Caspase 8 | GGGGTGCGACAACTATGACA | TGGCCCACTCTTCTGTTCAC |
|  | Caspase 9 | TGAGCCAGCCAGATGAACAG | GATATCCCCTGTGGCATCCC |
|  | Cytochrome oxydase Sub1 | GCTTTCTGTTGAGGTCGGGA | ACGCATGTTAAGCAGCGTGA |
|  | DIABLO | TTGTTCAGTCGGTCTCCTGC | AAGCACGCCAAACTAAGCAG |
|  | FAS2 | GCTACCCTTCGAACCAAGAG | GACTGTATCCCTCCCAGATGC |
|  | TNFα | GTCGTCTGCTCAATTCGCCT | TCGGCTAGATAAGGGCTTTTCC |
| *Lo. orbiculatus* | BAX | ATCGGCAGTTCTCCCAATCC | TCGACCATCTTCTCCATCGC |
|  | Bcl2-1 | ATGTAACTTCGGGATGGAGGAC | ACTACTATGACTCGACCCCAGT |
|  | Bcl2-2 | ACTGGCCACGAGTTATGTCATT | TCTTCAGGTGAGACATCTTGCC |
|  | Bcl2-3 | GCCGTTCAATGTGCTGAGAATT | TTCGTAGCCCTTCCATTCCATC |
|  | Caspase 3 | GGCATGTCAGGGTGTACAGTTA | TGCTACTTTCTTGTTGACCCGA |
|  | Caspase 7 | AATGCTGACACTGATGACGAGT | TTCGCTATGAACCAAGAACCGA |
|  | Caspase 8 | GGGGTGCGACAACTATGACA | TGGCCCACTCTTCTGTTCAC |
|  | Caspase 9 | CAAACTTACGTCTCTCAAGCGC | TCATTGTCACATTGCAACGACG |
|  | Cytochrome oxydase Sub1 | GCTTTCTGTTCAGGTCGGGA | ACGCATGTTAAGCAGGGTGA |
|  | DIABLO | TTGTTCAGTCGGTCTCCTGC | AAGCACGCCAAACTAAGCAG |
|  | FAS2 | GACTGTATCCCTCCCAGATGG | GCTACCCTTCGAACCAAGAGT |
|  | TNFα | GCAAAACTGGTGGATGCCTG | GCTTCAATGTCACCTTCGCC |


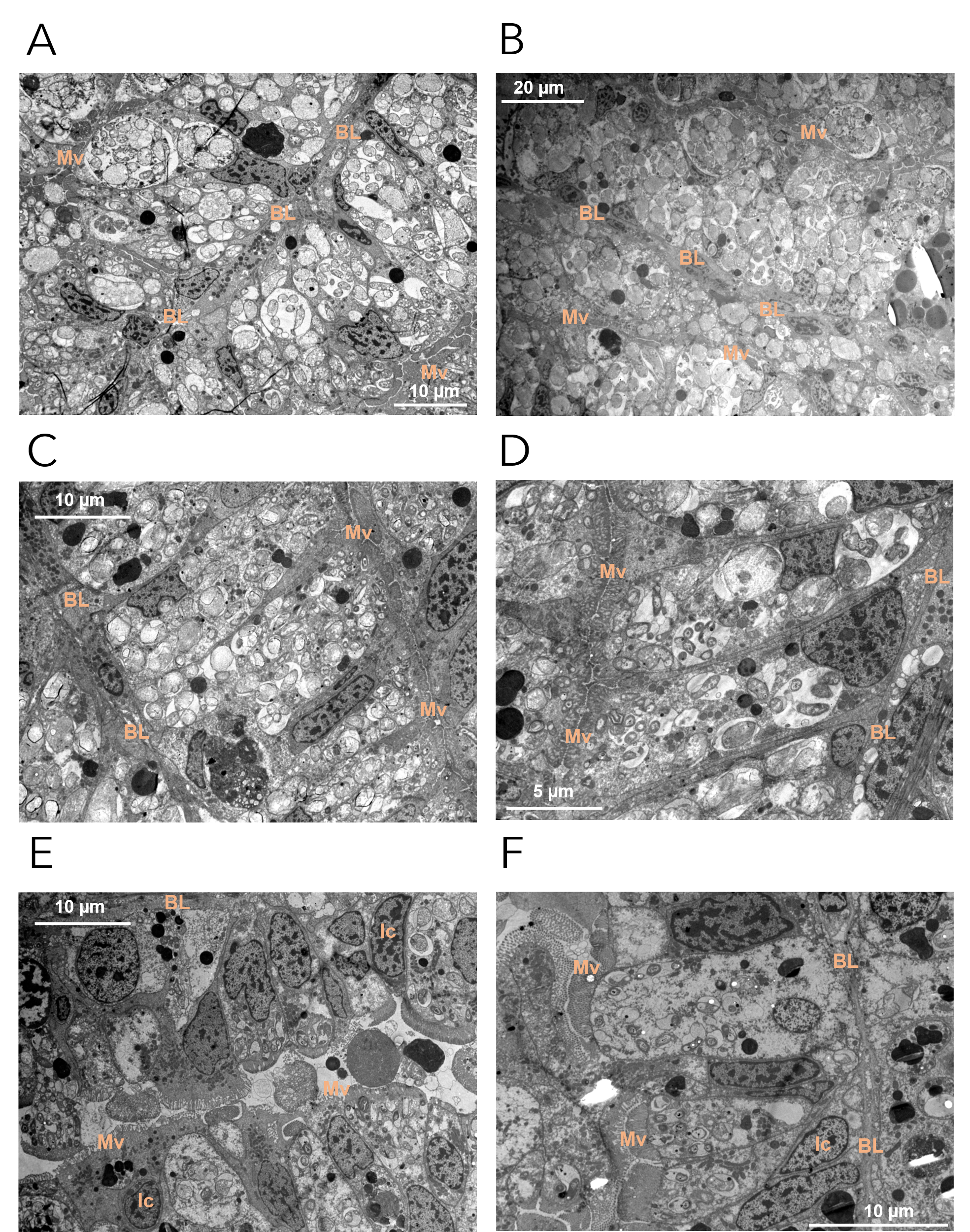


**Supplementary_figure_S1:** Gill epithelium ultrastructure of *Lu. borealis*. (A-C-E) Epithelium of a second specimen respectively at T0, T30 and T120. (B-D-F) Epithelium of a third specimen respectively at T0, T30 and T120. These pictures complement observations displayed of figure 1 on two additional specimens. Sulfide starvation induces modification and the alteration of the gill epithelium as well as the reduction of the symbiont’s density within the bacteriocytes. Bacteriocytes are oriented from the basal pole anchored in the basal lamina (BL) to the apical pole where are located the microvilli (Mv). During the starvation, some other cells appear like intercalary cells (Ic).


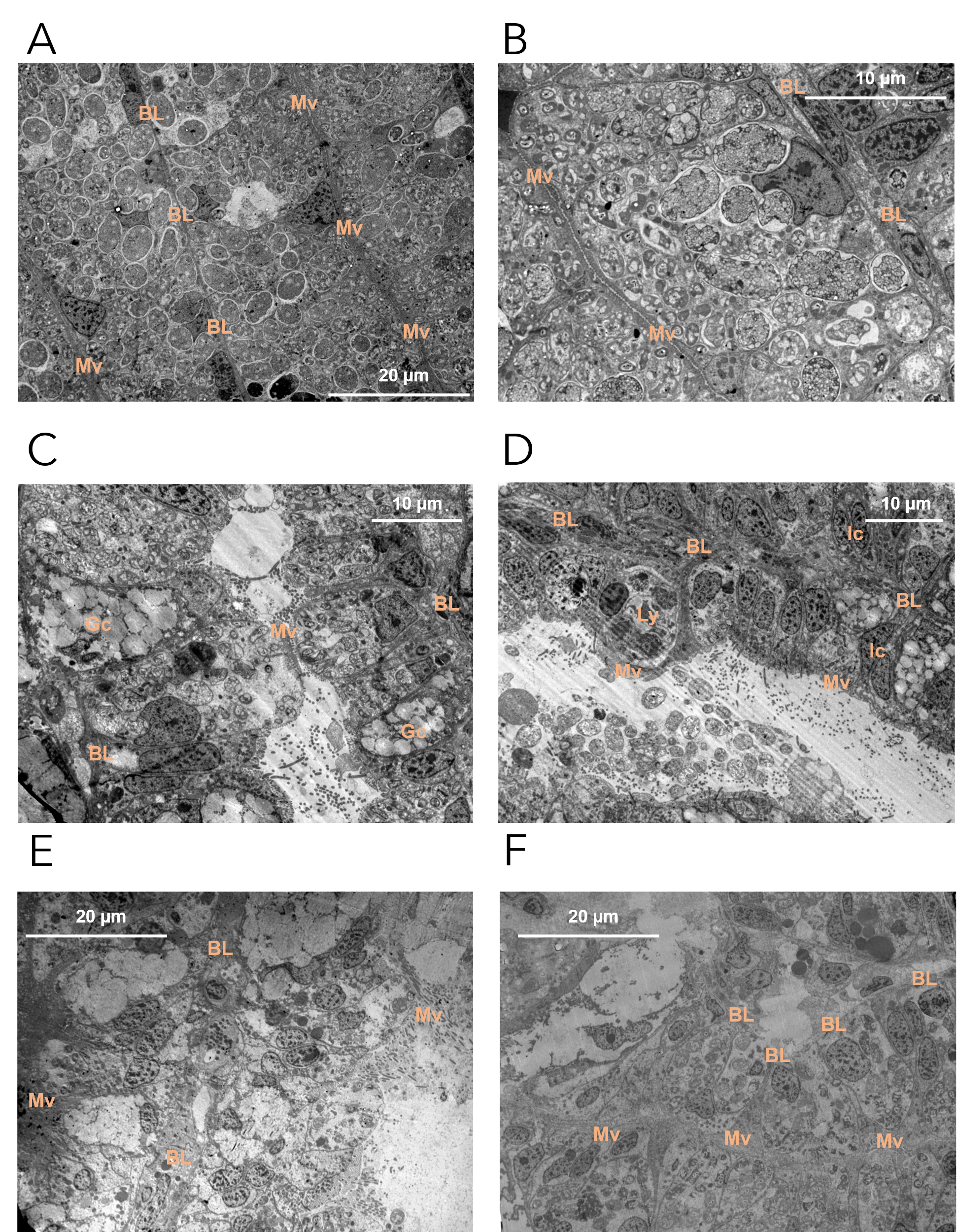


**Supplementary_figure_S2:** Gill epithelium ultrastructure of *Lo. orbiculatus*. (A-C-E) Epithelium of a second specimen respectively at T0, T30 and T120. (B-D-F) Epithelium of a third specimen respectively at T0, T30 and T120. These pictures complement observations displayed of figure 1 on two additional specimens. Sulfide starvation induces modification and the alteration of the gill epithelium as well as the reduction of the symbiont’s density within the bacteriocytes. Bacteriocytes are oriented from the basal pole anchored in the basal lamina (BL) to the apical pole where are located the microvillosity (Mv). During the starvation, some other cells appear like intercalary cells (Ic) as well as more numerous goblet cells (Gc) in *Lo. orbiculatus*.


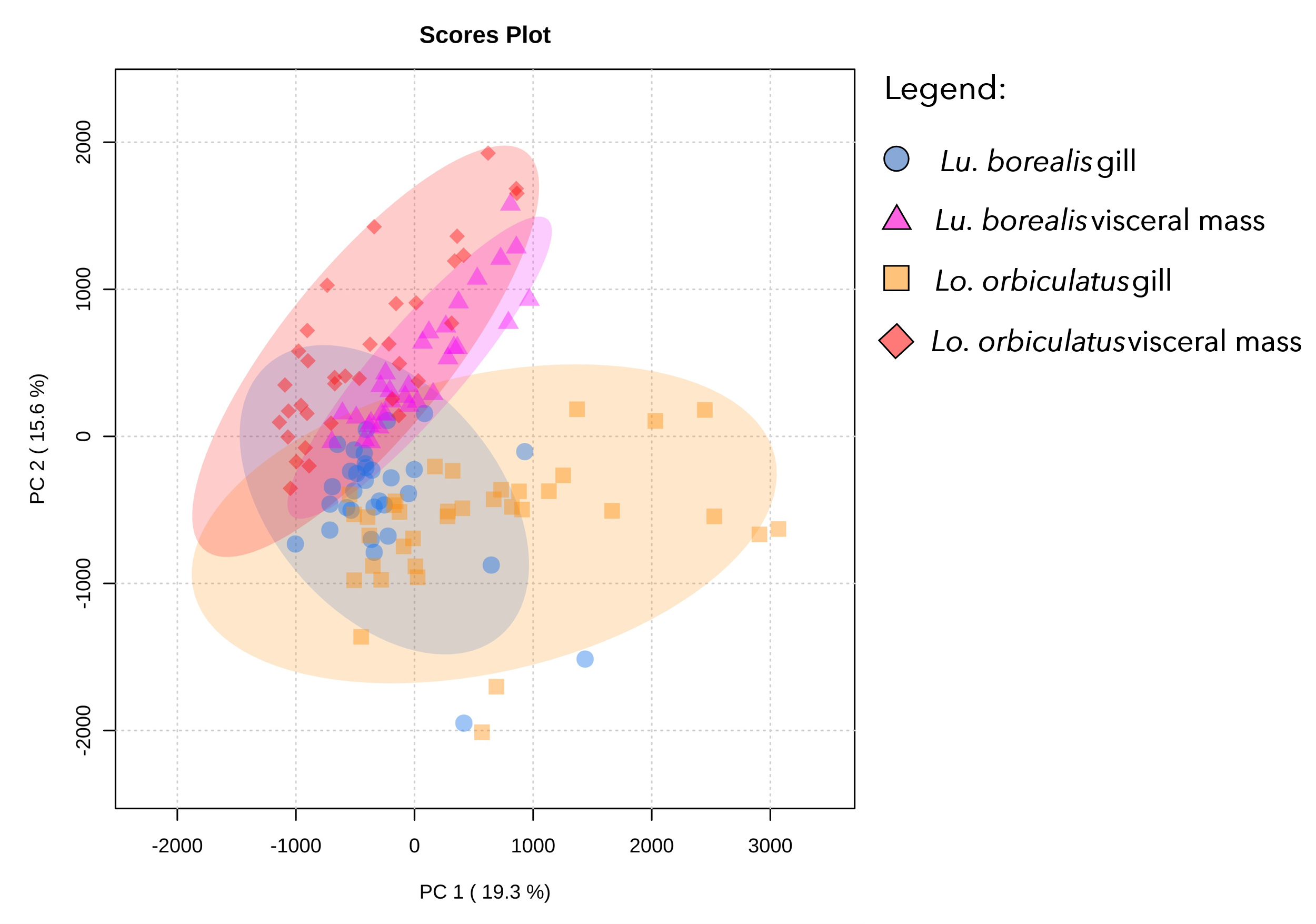


**Supplmentary_figure_S3:** PCA of the metabolome composition of Loripes orbiculatus and Lucinoma borealis gill and visceral mass throughout the experiment.


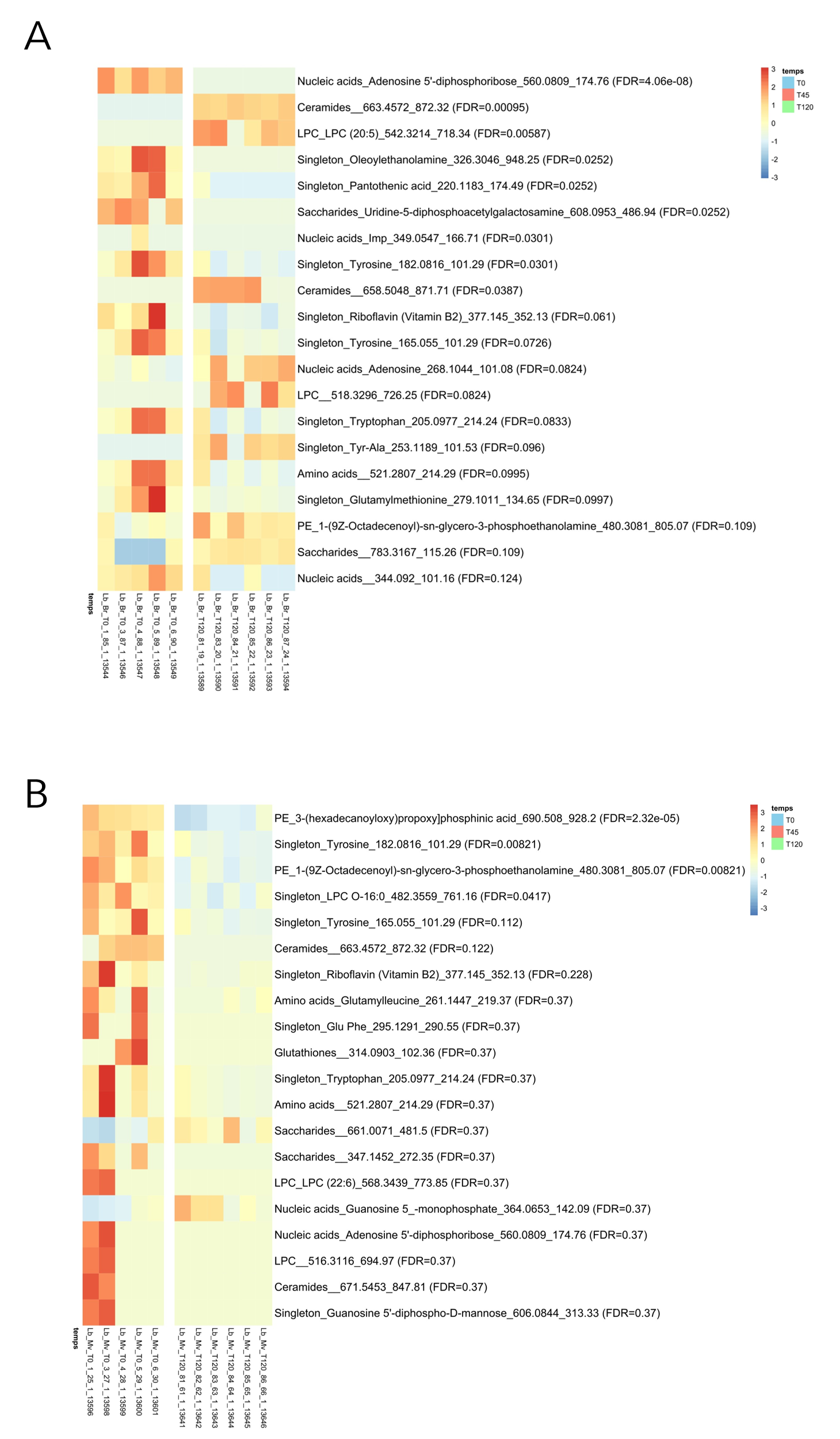

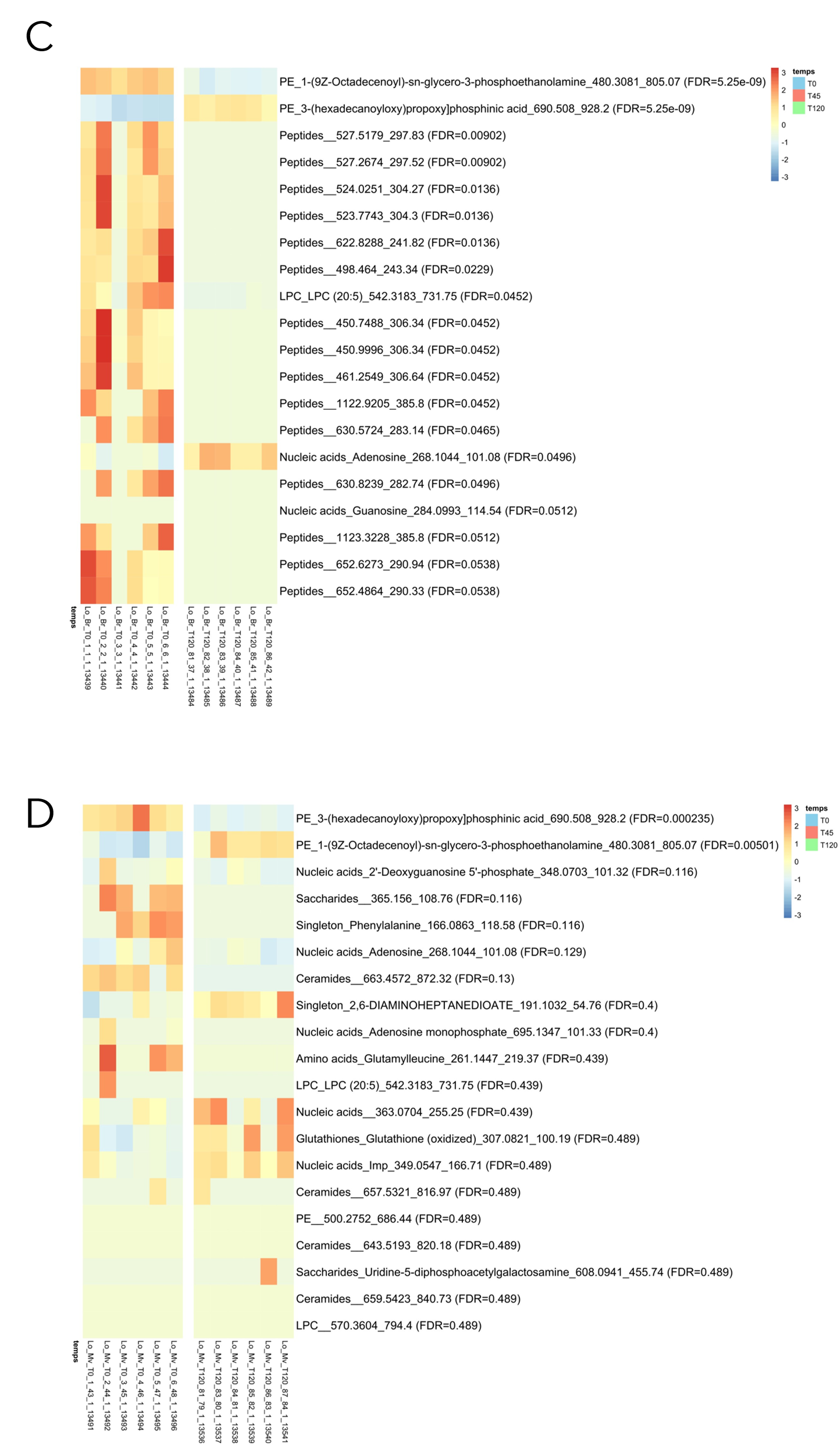


**Supplementary_figure_S4:** Heatmap of the top 20 most discriminant metabolites explaining variations in metabolomic profiles in the two Lucinidae species over time. (A) Most discriminant metabolites in the gill of Lucinoma borealis during the four-month sulfide deprivation period. (B) Most discriminant metabolites in the visceral mass of Lu. borealis during the four-month sulfide deprivation period. (C) Most discriminant metabolites in the gill of Loripes orbiculatus during the four-month sulfide deprivation period. (D) Most discriminant metabolites in the visceral mass of Lo. orbiculatus during the four-month sulfide deprivation period. Discriminant metabolites were identified using ANOVA followed by Tukey’s post hoc test with FDR-adjusted p-values.
